# Waist circumference mediates the relationship between atherogenic index of plasma and infertility

**DOI:** 10.3389/fendo.2025.1473228

**Published:** 2025-03-20

**Authors:** Xue Wei, Dandan Liu

**Affiliations:** Department of Endocrinology, The Eighth Affiliated Hospital of Sun Yat-sen University, Sun Yat-sen University, Shenzhen, China

**Keywords:** atherogenic index of plasma, infertility, NHANES, waist circumference, cross-sectional study

## Abstract

**Background:**

A newly developed technique, Atherogenic Index of Plasma (AIP), is linked to numerous metabolic disorders. Prior researches have indicated strong correlation between AIP and waist circumference (WC), as well as between WC and infertility. Yet no investigation has examined link involving the AIP and infertility, as well as the potential mediating role of WC in this relationship.

**Methods:**

The study included 1,322 women from the 2013–2018 NHANES. Infertility was the outcome variable. Moreover, mediation analysis explored the mediating role of WC in the above relationships.

**Results:**

There were 1,163 controls and 159 infertile participants among the 1,322 participants. The study demonstrated increased WC and elevated AIP among infertile women. Also, the AIP demonstrated an independent correlation with a higher likelihood of infertility, regardless of adjustments for confounding factors. Subgroup analysis indicated the AIP was related to the prevalence of infertility even among women aged 35 years or younger with no history of cardiovascular disease (CVD), pelvic infections, or use of female hormones. Finally, WC had a substantial mediating effect on correlation between AIP and infertility, accounting for 54.49% of the association. Yet, it appears that the various IR surrogates did not demonstrate variability in their predictive ability for infertility [AIP: 0.642 (95% CI: 0.599, 0.683) vs. WC 0.658 (95% CI: 0.618, 0.705) vs. HOMA-IR 0.637 (95% CI: 0.593, 0.686)].

**Conclusion:**

A notable positive correlation exists between AIP and female infertility. It provides the first evidence to demonstrate the mediating role of WC in the above relationship. Managing abdominal obesity and monitoring AIP levels may contribute to reduce the likelihood of infertility.

## 1 Introduction

An estimated 8 to 12% of couples worldwide experience infertility, which is characterized as the inability to conceive after 12 months of regular, unprotected intercourse (1–3). The infertility rate among women in the US aged 18 to 45 has been rising, increasing from 5.8% in the period 2006-2010 to 8.1% in 2017-2019 (4). Beyond its emotional toll, infertility precipitates significant socio-economic challenges and familial strain, positioning it as a pressing public health issue. Consequently, the U.S. Centers for Disease Control and Prevention (CDC) underscores the importance of prioritizing the diagnosis and treatment of infertility to mitigate its impact (5).

An expanding body of both animal and clinical research underscores a notable correlation between infertility and insulin resistance (IR) (6–8). A widely utilized and straightforward way to measure IR is the Homeostasis Model Assessment of Insulin Resistance (HOMA-IR), yet it is challenging to implement in the vast majority of areas that are developing. AIP, a novel, straightforward, and dependable marker for atherosclerosis and cardiovascular disease risk prediction, is derived from the logarithmic transformation of the triglyceride (TG) to high-density lipoprotein cholesterol (HDL-C) ratio (9–15). Recent investigations have established a robust correlation between AIP and conditions such as IR, prediabetes, and diabetes, highlighting its potential as a significant predictor of metabolic disorders (16–20). Given the pronounced link between AIP and IR, exploring the potential connection between AIP and infertility emerges as a crucial step towards identifying more convenient and accessible markers for screening infertility in women of reproductive age. Yet, to date, an investigation into the relationship between AIP and the prevalence of infertility among reproductive-aged females remains unexplored.

Previous studies have identified WC as a contributing factor to infertility (21–23). Additionally, prior researches have indicated strong correlations between AIP and WC (24–26). Nevertheless, the interaction between them remains thoroughly unexplored. Exploring the mediating role of WC could offer valuable insights into the mechanisms by which AIP impacts the risk of infertility, potentially uncovering new avenues for intervention. This could lead to more targeted interventions, ultimately improving infertility prevention.

Thus, we endeavor to illuminate the correlation between the AIP and infertility in the US by conducting a cross-sectional investigation of information from the NHANES covering the years 2013 to 2018.

## 2 Materials and methods

### 2.1 Study design and participants

**Figure 1:**
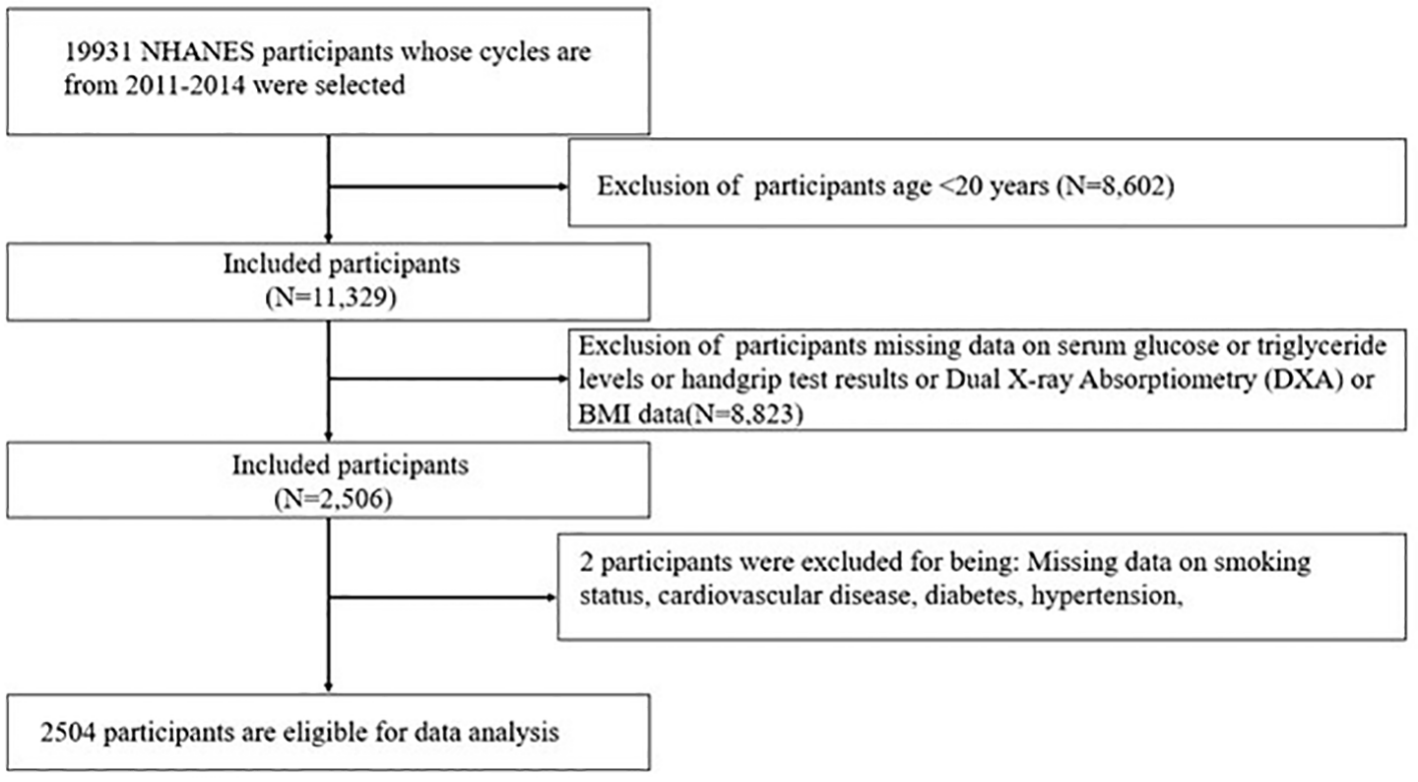
Flowchart for selecting samples.

The NHANES was conducted under the oversight of the NCHS Research Ethics Review Board. This investigation utilized data from the 2013–2018 NHANES, encompassing a cohort of 29,400 participants. The study’s methodology is depicted in **Figure 1**. The exclusion criteria were rigorously applied as follows: (1) 14451 male participants were excluded; (2) 10,625 individuals beyond the age of 45 years or younger than 18 years were omitted; (3) those without available AIP values were excluded (N=2,574); (4) subjects with incomplete fertility data were removed (N=194); (5) participants missing either the outcome of interest or necessary covariate information were also excluded (N=234). Following this meticulous screening process, a cohort of 1,322 eligible subjects was retained for subsequent analysis.

### 2.2 Assessment of infertility

Using self-reports and the reproductive health questionnaire, infertility was characterized. Individuals who affirmed experiencing infertility were classified into the infertility group; otherwise, they were categorized as not having infertility. To maintain the integrity and precision of the study’s outcomes, participants who either abstained from answering or expressed uncertainty about their fertility status were systematically removed from the analysis. This approach ensured that the analysis was conducted with the most accurate and reliable data available.

### 2.3 Calculation of AIP

The formula for calculating AIP which is an exposure variable, is log [TG (mg/dL)/HDL-C(mg/dL)]. Utilizing subsequent definitions of the quartiles, subjects were divided into different categories relying on their AIP values: Q1 (AIP < -0.077), Q2 (AIP -0.077 to <0.127), Q3 (AIP -0.127 to < 0.348), and Q4 (AIP ≥ 0.348) (27).

### 2.4 Assessment of waist circumference and HOMA-IR

All waist circumference is taken at the mobile examination center (MEC). HOMA-IR is equal to fasting insulin (µU/mL) ×fasting glucose (mmol/L)/22.5 (28).

### 2.5 Selection of covariables

In this study, covariates were meticulously selected to encompass both continuous variables, such as age and poverty income ratio (PIR), and categorical variables. The categorical variables included race, smoking status, education level, BMI status, CVD, leisure time physical activity (LTPA), daily sitting time, history of treatment for pelvic inflammatory disease (PID), usage of female hormones, and history of taking birth control pills in accordance with prior characterized or clinical expertise (21–23).

The categorizations of participants’ responses regarding PID, whether they have ever used birth control pills, and whether they have ever used female hormones are straightforwardly determined based on participants’ responses, categorized as either “yes” or “no”. CVD encompassed conditions that were clinically diagnosed, including coronary heart disease, congestive heart failure, stroke, and angina. Self-reported data on daily sitting time and weekly LTPA were collected through respondent-level interviews. Based on their sedentary time, participants were classified into four groups (29). For physical activity levels, participants were divided into three categories: inactive (individuals not engaging in any LTPA), insufficiently active (0 - 150 min LTPA/week), and sufficiently active (>150 min LTPA/week) (30).

### 2.6 Statistical analysis

The statistical analyses were conducted using R 4.4.2 software and EmpowerStats. The threshold for determining statistical significance was established at *P* < 0.05. Characteristics across groups were contrasted utilizing t-tests and chi-square tests in **Table 1**. Levels of AIP were stratified into four groups for analysis. This analysis involved the calculation of odds ratios (ORs) and 95% confidence intervals (CIs) to quantify the strength and precision of these associations. No factor was altered by Model 1. Model 2 brought into aspects including age, racial background, levels of education, and PIR. Model 3 was adjusted for smoked status, pelvic infection, ever taken birth control pills, ever use female hormones, CVD, daily sitting time, LTPA, building upon the adjustments made in Model 2. Subsequently, the effects of AIP on infertility were evaluated through subgroup analyses and interaction tests, focusing on groups characterized by age, smoked status, daily sitting time, history of pelvic infection, usage of female hormones, history of taking birth control pills, CVD and LTPA.

**Table 1:** The demographic and clinical characteristics of participants by quartiles of baseline AIP.

Variable	Q1(<-0.077)	Q2(-0.077 to <0.127)	Q3(0.127 to <0.348)	Q4(≥ 0.348)	*P* value
**Participants**	331	330	330	331	
**Age, year**	31.57 ± 7.12	32.52 ± 7.58	32.55 ± 7.55	34.00 ± 7.51	**<0.001**
**PIR**	2.57 ± 1.62	2.34 ± 1.52	2.06 ± 1.50	2.08 ± 1.45	**<0.001**
**WC**	85.87 ± 14.08	92.76 ± 17.78	99.88± 18.61	105.88± 16.99	**<0.001**
**Race, N(%)**					**<0.001**
Mexican American, N(%)	34 (10.27%)	44 (13.33%)	64 (19.39%)	82 (24.77%)	
Other Hispanic, N(%)	28 (8.46%)	28 (8.48%)	35 (10.61%)	43 (12.99%)	
Non-Hispanic white, N(%)	98 (29.61%)	113 (34.24%)	121 (36.67%)	121 (36.56%)	
Non-Hispanic black, N(%)	102 (30.82%)	83 (25.15%)	65 (19.70%)	26 (7.85%)	
Non-Hispanic Asian, N(%)	61 (18.43%)	46 (13.94%)	26 (7.88%)	38 (11.48%)	
Other race, N(%)	8 (2.42%)	16 (4.85%)	19 (5.76%)	21 (6.34%)	
**Education level, N(%)**					**<0.001**
Less than 9th grade, N(%)	12 (3.63%)	9 (2.73%)	26 (7.88%)	26 (7.85%)	
9th–11th grade, N(%)	26 (7.85%)	26 (7.88%)	36 (10.91%)	50 (15.11%)	
High school graduate/GED or equivalent, N(%)	52 (15.71%)	64 (19.39%)	65 (19.70%)	77 (23.26%)	
Some college or AA degree, N(%)	113 (34.14%)	126 (38.18%)	121 (36.67%)	111 (33.53%)	
College graduate or above, N(%)	128 (38.67%)	105 (31.82%)	82 (24.85%)	67 (20.24%)	
**Smoked status, N (%)**					**<0.001**
No, N(%)	249 (75.23%)	248 (75.15%)	219 (66.36%)	211 (63.75%)	
Yes, N(%)	82 (24.77%)	82 (24.85%)	111 (33.64%)	120 (36.25%)	
**CVD, N(%)**					0.424
No, N(%)	324 (97.89%)	324 (98.18%)	323 (97.88%)	319 (96.37%)	
Yes, N(%)	7 (2.11%)	6 (1.82%)	7 (2.12%)	12 (3.63%)	
**Daily sitting time (h/day), N(%)**					0.685
<4	112 (33.84%)	130 (39.39%)	115 (34.85%)	121 (36.56%)	
4–<6	71 (21.45%)	78 (23.64%)	67 (20.30%)	70 (21.15%)	
6–8	73 (22.05%)	54 (16.36%)	67 (20.30%)	65 (19.64%)	
**>=**8	75 (22.66%)	68 (20.61%)	81 (24.55%)	75 (22.66%)	
**LTPA, N(%)**					
Inactive	119 (35.95%)	130 (39.39%)	163 (49.39%)	178 (53.78%)	**<0.001**
Insufficiently active	52 (15.71%)	61 (18.48%)	64 (19.39%)	59 (17.82%)	
Physically active	160 (48.34%)	139 (42.12%)	103 (31.21%)	94 (28.40%)	
**Ever treated for a pelvic infection/PID, N(%)**					0.745
No, N(%)	316 (95.47%)	317 (96.06%)	314 (95.15%)	312 (94.26%)	
Yes, N(%)	15 (4.53%)	13 (3.94%)	16 (4.85%)	19 (5.74%)	
**Ever taken birth control pills, N(%)**					0.082
No, N(%)	118 (35.65%)	92 (27.88%)	113 (34.24%)	96 (29.00%)	
Yes, N(%)	213 (64.35%)	238 (72.12%)	217 (65.76%)	235 (71.00%)	
**Ever use female hormones, N(%)**					0.282
No, N(%)	324 (97.89%)	315 (95.45%)	319 (96.67%)	316 (95.47%)	
Yes, N(%)	7 (2.11%)	15 (4.55%)	11 (3.33%)	15 (4.53%)	
**Infertility**					**0.006**
No, N(%)	307 (92.75%)	292 (88.48%)	278 (84.24%)	286 (86.40%)	
Yes, N(%)	24 (7.25%)	38 (11.52%)	52 (15.76%)	45 (13.60%)	

WC waist circumference, PIR poverty income ratio, CVD cardiovascular disease, LTPA leisure time physical activity.

*P* in bold indicates a statistically significant difference.

In addition, the non-linear connections between AIP and infertility were researched utilizing a Generalized Additive Model (GAM). Furthermore, mediation analysis was carried out to investigate whether waist circumference mediated the relation between AIP and infertility in reproductive-aged women.

## 3 Results

### 3.1 Baseline characteristics of the participants

There were 1,163 controls and 159 infertile participants among the 1,322 participants. **Table 1** reveals the features of the subjects categorized by AIP quartiles. AIP levels failed to indicate any meaningful correlations with CVD, daily sitting duration, history of pelvic infection, usage of female hormones, or history of taking birth control pills (*P* > 0.05). In contrast, AIP were correlations with to age, race, education level, PIR, WC, smokers, LTPA and individuals with infertility (all P-values < 0.05). Participants with increased AIP tended to be older with a larger WC, lower PIR, and education levels compared to those in the lower AIP quartile groups.

### 3.2 Association between AIP and infertility

The results demonstrated a considerably greater likelihood of WC and elevated AIP among infertile women in the US, across all models, regardless of adjustments for confounding factors in **Table 2**. After adjusting for all confounding variables (model 3), each 1-unit increase in AIP was linked with an 94% increase in the odds of infertility. Furthermore, in Model 3, which adjusted for all relevant covariates, participants in the highest AIP quartile had an OR of 1.97(95% CI: 1.13–3.44), indicating an 97% higher likelihood of infertility in comparison with others in the lower AIP quartile groups.

**Table 2:** Relative odds of Infertility according to AIP in different models among all participants.

	Model 1 OR (95% CI, *P*)	Model 2 OR (95% CI, *P*)	Model 3 OR (95% CI, *P*)
Continuous variables			
AIP	2.00(1.22–3.27) **0.006**	1.89(1.11–3.22) **0.018**	1.94 (1.13–3.33) **0.016**
Categorical variable			
Q1	Ref	Ref	Ref
Q2	1.66(0.97–2.84) 0.062	1.59(0.92–2.74) 0.093	1.63(0.94–2.81) 0.082
Q3	2.39(1.44–3.99) **<0.001**	2.36(1.40–3.99) **0.001**	2.47(1.45–4.20) **<0.001**
Q4	2.01(1.20–3.39) **0.008**	1.91(1.10–3.30) **0.021**	1.97(1.13–3.44) **0.017**
***P* for trend**	0.007	0.018	0.015

Model 1: not adjusted for any covariables.

Model 2: adjusted for age, race, education level and PIR.

Model 3: adjusted for all relevant covariates including age, race, education level, PIR, smoked status, pelvic infection, ever taken birth control pills, ever use female hormones, CVD, daily sitting time, LTPA.

OR odds ratio, CI confidence interval, Ref reference.

*P* in bold indicates a statistically significant difference.

### 3.3 Non-linear relationships

**Figure 2:**
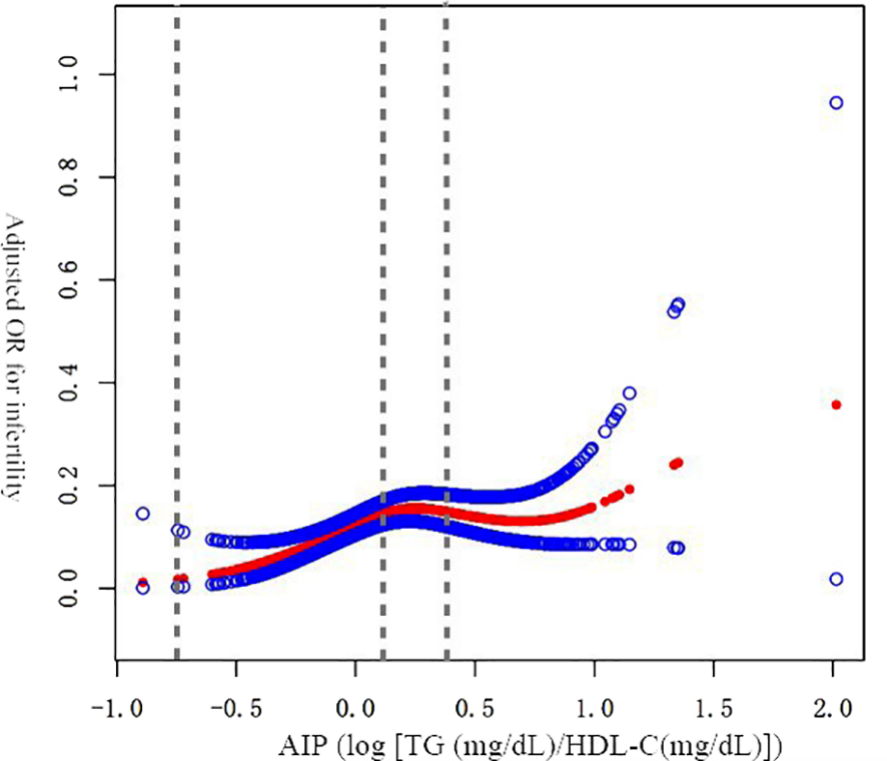
Non-linear link between the AIP and infertile participants. adjusted for all relevant covariates including adjusted for age, racial background, levels of education, PIR, smoked status, PID, ever taken birth control pills, ever use female hormones, CVD, daily sitting time, LTPA.

The smooth curve fitting demonstrated a non-linear link between AIP and infertile participants (**Figure 2**). Further calculations determined the inflection point to be 0.16 (**Table 3**). AIP and female infertility exhibited an intensely favorable relationship before the inflection point, with an OR of 11.13(95% CI: 2.83–43.72). This indicates a strong relationship between higher AIP levels and increased infertility risk up to the inflection point. However, beyond the inflection point, the link between AIP and female infertile participants was not significant, with an OR of 0.71(95% CI: 0.28–1.78), indicating a significant threshold effect of AIP and infertility.

**Table 3:** Threshold effect analysis of AIP on female infertility using a two-piecewise linear regression model.

	Adjusted OR (95% CI)	*P* value
AIP	1.94(1.13–3.33)	**0.016**
Inflection point 1		
< 0.16	11.13(2.83–43.72)	< **0.001**
> 0.16	0.71(0.28–1.78)	0.464
Log-likelihood ratio	0.003	

OR odds ratios, CI confidence interval, ref reference group.

*P* in bold indicates a statistically significant difference.

### 3.4 Subgroup analyses

To further investigate the factors influencing the link between AIP and the likelihood of female infertility, we conducted stratified analyses and interaction tests based on age, smoked status, daily sitting time, history of pelvic infection, usage of female hormones, history of taking birth control pills, CVD and LTPA (**Table 4**). Significant interactions were detected for age, smoked status, daily sitting time, history of pelvic infection, ever taken birth control pills, ever use female hormones, CVD and LTPA, with all *P*-values for interaction being < 0.05. A positive link between AIP and female infertile participants persisted in specific subgroups, including women aged 35 years or younger, smokers, people whether have CVD, those without a history of pelvic infections, users of female hormones, and individuals who have ever taken birth control pills. Notably, the subgroup analysis indicated that even among women aged 35 years or younger with no history of cardiovascular disease, pelvic infections, or use of female hormones, an elevated AIP still correlates with the gradually relative odds of infertility.

**Table 4:** Stratified associations between AIP and female infertility according to baseline characteristics.

Subgroup	N	Adjusted OR (95% CI)	*P* value	*P* for interaction
**Age**				**0.014**
<35	803	4.16 (1.81, 9.56)	**<0.001**	
≥35	519	1.09 (0.51, 2.34)	0.821	
**Smoked status, N (%)**				**0.016**
No, N(%)	927	1.70 (0.84, 3.41)	0.138	
Yes, N(%)	395	2.76 (1.14, 6.71)	**0.025**	
**CVD, N(%)**				**0.016**
No, N(%)	1290	1.90 (1.10, 3.41)	**0.021**	
Yes, N(%)	32	0.00 (0.00, 0.00)	**<0.001**	
**Daily sitting time**				**0.013**
<4	478	0.84 (0.30, 2.34)	0.743	
4–<6	286	3.53 (0.89, 13.99)	0.073	
6–<8	259	3.60 (0.94, 13.82)	0.062	
≥8	299	1.73 (0.57, 4.25)	0.334	
**LTPA, N(%)**				**0.038**
Inactive	590	2.11 (0.94, 4.77)	0.072	
Insufficiently active	236	1.58 (0.39, 6.36)	0.523	
Physically active	496	1.68 (0.64, 4.43)	0.292	
**Ever treated for a pelvic infection/PID, N(%)**				**0.016**
No, N(%)	1259	1.84 (1.05, 3.23)	**0.034**	
Yes, N(%)	63	0.97 (0.04, 26.19)	0.984	
**Ever taken birth control pills, N(%)**				**0.016**
No, N(%)	419	1.74 (0.59, 5.15)	0.314	
Yes, N(%)	903	2.10 (1.10, 3.99)	**0.024**	
**Ever use female hormones, N(%)**				**0.016**
No, N(%)	1274	1.83 (1.05, 3.20)	**0.032**	
Yes, N(%)	48	0.08 (0.00,2876.1)	0.633	

OR odds ratios, CI confidence interval, ref reference group.

*P* in bold indicates a statistically significant difference.

### 3.5 Association between the waist circumference and infertile participants

All of the models in **Table 5** demonstrated an important positive correlation between WC and female infertility [model 1: OR (95% CI) =1.020(1.011–1.028); model 2: OR (95% CI) = 1.017(1.008–1.027); model 3: OR (95% CI) = 1.018 (1.009–1.028)].

**Table 5:** Waist circumference and female infertility.

	Model 1 OR (95% CI, *P*)	Model 2 OR (95% CI, *P*)	Model 3 OR (95% CI, *P*)
Continuous	1.020 (1.011–1.028) **<0.001**	1.017 (1.008–1.027) **<0.001**	1.018 (1.009–1.028) **<0.001**

Model 1: not adjusted for any covariables.

Model 2: adjusted for age, race, education level and PIR.

Model 3: adjusted for age, racial background, levels of education, PIR, smoked status, pelvic infection, ever taken birth control pills, ever use female hormones, CVD, daily sitting time, LTPA. OR, odds ratio; CI, confidence interval. *P* in bold indicates a statistically significant difference.

### 3.6 Association between the AIP and waist circumference

An increased AIP level was associated with an elevated risk of WC across all models [model 1: β (95% CI) = 16.14(20.38–26.02); model 2: β (95% CI) = 16.37(20.56–26.15); model 3: β (95% CI) = 5.60 (19.79–25.48)] as shown in **Table 6**. In Model 3, the WC of the highest quartile was 14.6cm higher than that of the lowest quartile.

**Table 6:** Relative odds of waist circumference according to AIP in different models among all participants.

	Model 1 OR (95% CI, *P*)	Model 2 OR (95% CI, *P*)	Model 3 OR (95% CI, *P*)
Continuous variables			
AIP	16.14 (20.38–26.02) **<0.001**	16.37 (20.56–26.15) **<0.001**	15.60 (19.79–25.48) **<0.001**
Categorical variable			
Q1	Ref	Ref	Ref
Q2	5.22 (4.30–9.47) **<0.001**	4.91 (3.66–8.52) **<0.001**	4.82 (3.55–8.43) **<0.001**
Q3	10.62 (11.42–16.59) **<0.001**	10.21 (10.41–15.36) **<0.001**	9.77 (9.94–14.93) **<0.001**
Q4	15.19 (17.42–22.59) **<0.001**	15.22 (17.17–22.25) **<0.001**	14.60 (16.55–21.68) **<0.001**

Model 1: not adjusted for any covariables.

Model 2: adjusted for age, race, education level and PIR.

Model 3: adjusted for all relevant covariates including age, race, education level, PIR, smoked status, pelvic infection, ever taken birth control pills, ever use female hormones, CVD, daily sitting time, LTPA. OR, odds ratio; CI, confidence interval. *P* in bold indicates a statistically significant difference.

### 3.7 Mediating effect of waist circumference on AIP and infertility

**Figure 3:**
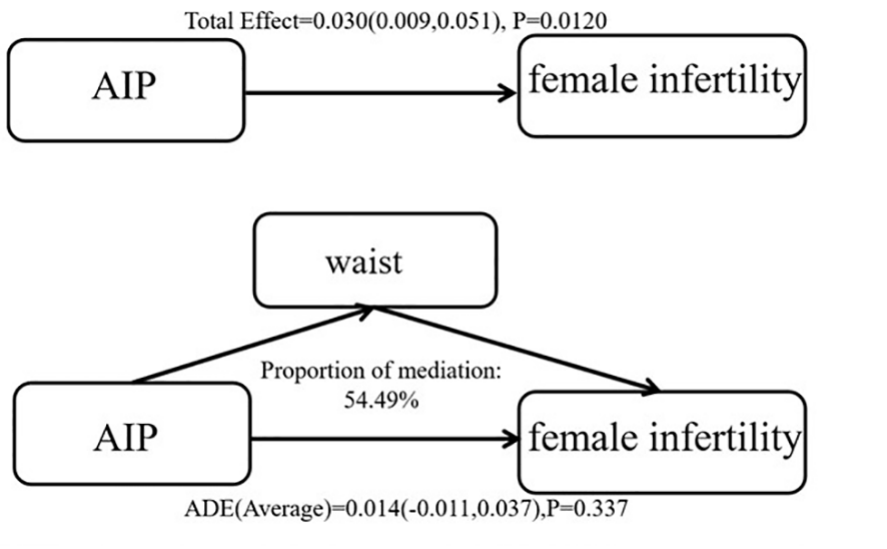
Mediation analysis of hypertension on the risk of AIP and infertility.

As depicted in **Figure 3**, WC demonstrated a strong link with infertility [total effect (95% CI, *P*): 0.030(0.009,0.051), 0.012]. Additionally, there was a substantial moderating effect for WC in the link between AIP and infertility [mediation effect (95% CI, *P*): 0.016(0.005–0.026), 0.004], accounting for 54.49% of the association. While there was no direct correlation between AIP and infertility [direct effect (95% CI, *P*): 0.014 (-0.011, 0.037), 0.337]. These findings indicate that WC served as a significant complete mediator in the link between AIP and infertility.

### 3.8 Comparison of different IR surrogates in predicting infertility

**Figure 4:**
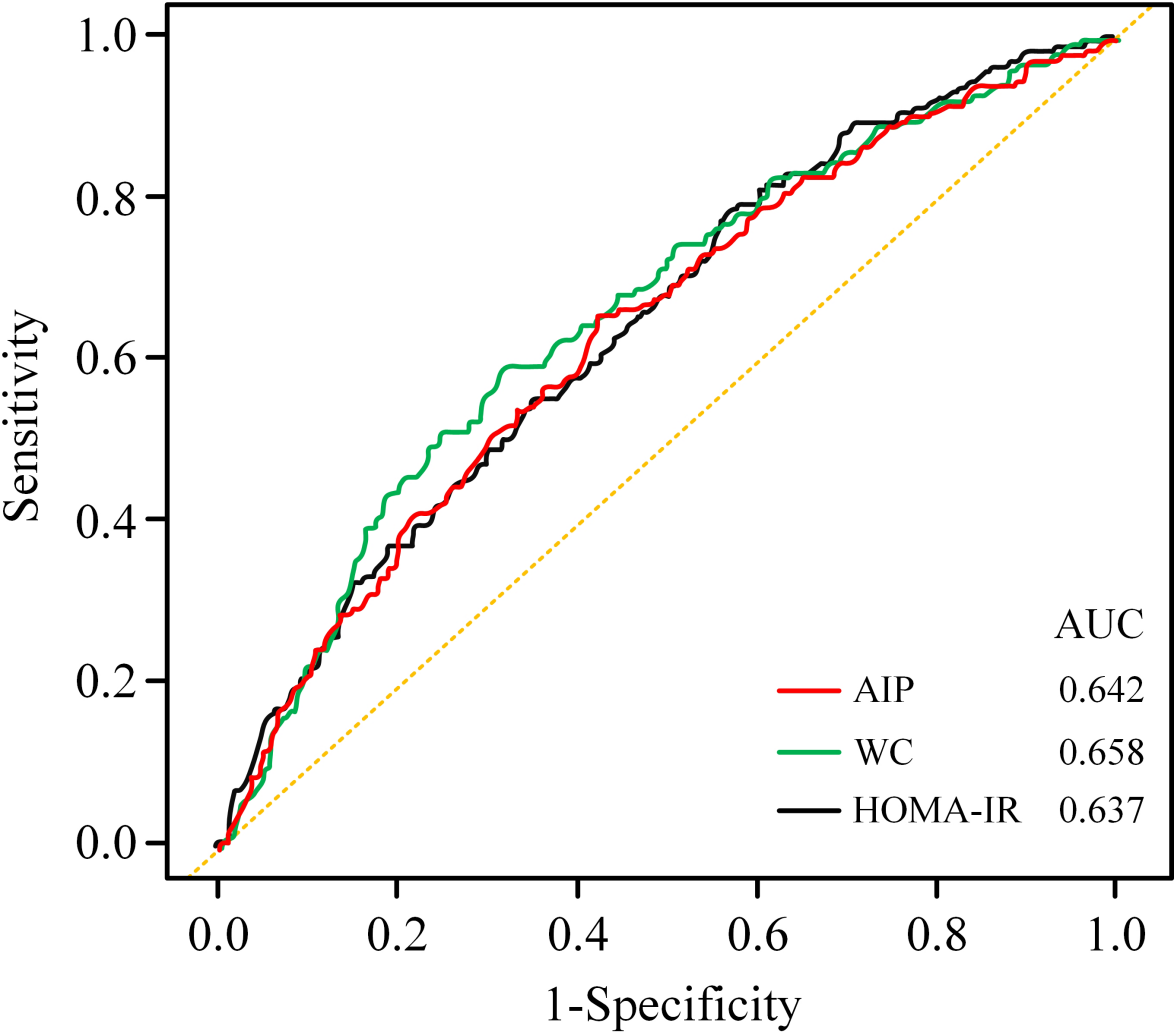
Comparison of ROC curves for different surrogates to predict infertility.

The results of the ROC curves were presented in **Table 7**; **Figure 4**. Regarding infertility, the AUCs of AIP, WC and the HOMA-IR index were 0.642 (95% CI: 0.599, 0.683) vs. 0.658 (95% CI: 0.618, 0.705) vs. 0.637 (95% CI: 0.593, 0.686). Although the AUC of WC was higher, there was no statistically significant difference between the AUCs of the diverse surrogates (*P* > 0.05). It appears that the various IR surrogates did not demonstrate variability in their predictive ability for infertility.

**Table 7:** Comparison of ROC curves for different surrogates to predict infertility.

Surrogates	Cutoff (Sensitivity, Specificity)	AUC (95% CI)	P-value
Infertility			
HOMA-IR index	14.478(0.554, 0.571)	0.637 (95% CI: 0.593, 0.686).	>0.05
WC	81.850(0.541, 0.667)	0.658 (95% CI: 0.618, 0.705)	
AIP	-0.021(0.818, 0.329)	0.642 (95% CI: 0.599, 0.683)	

## 4 Discussion

The study involving 1,322 women aged 18–45 years, demonstrated a positive correlation between AIP and experiencing infertility regardless of adjustments for confounding factors. A nonlinear correlation between AIP and infertility was observed, with different relationships detected on either side of the breakpoint (AIP = 0.16). Specifically, AIP was positively associated with the likelihood of infertility on the left side of the breakpoint, while the association on the right side was not statistically significant, indicating a significant threshold effect of AIP on female infertility. Significant associations between AIP and infertility were more likely to occur among women aged 35 years or younger with no history of cardiovascular disease, pelvic infections, or use of female hormones. Additionally, this research demonstrated that WC is a contributing factor to infertility, and high levels of AIP are positively correlated with WC. Notably, WC was a significant complete mediator of the relationship between AIP and infertility. Yet, it appears that the various IR surrogates did not demonstrate variability in their predictive ability for infertility.

Studies have shown that polycystic ovary syndrome (PCOS), endometriosis, and endometritis are linked to infertility. As of now, there have been no studies that have demonstrated a potential link between AIP and infertile participants. Yet Previous investigations demonstrate that lipid metabolism are associated with an increased risk of infertility. For instance, Essah et al. found that American women with PCOS had higher mean TG levels and lower mean serum HDL cholesterol levels (31). Similarly, Fatma et al. demonstrated that elevated TG and decreased HDL-C were linked to the exacerbation of endometriosis in women (32). Consistent with prior investigations, our study identified that elevated AIP have a correlation with a higher likelihood of infertility. Additionally, subgroup analyses showed that the above correlation was more pronounced in women aged 35 years or younger. This finding is intriguing as it challenges the common belief that advanced age is the primary factor contributing to reproductive dysfunction in women, but our study indicated otherwise. This may be related to estrogen in females, lowering TG level and influencing AIP. A separate study revealed that the serum estradiol levels were elevated in older women compared to younger women (21–35 years) (33, 34). The specific mechanism needs to be further studied.

Prior research has indicated that obesity has a negative impact on reproductive health, particularly in relation to infertility (35–39). As the prevalence of abdominal obesity increases, WC is crucial for obesity surveillance (40). Many research has demonstrated a correlation between WC and infertility. Li et al. demonstrated a negative relationship between WC and infertile women (21). The smaller WC was connected with a higher likelihood of becoming pregnant, according to research by Moran et al. (41). Furthermore, in a cross-sectional observational study of 3239 women in American, Jierong et al. observed that WC, was relevant in predicting female infertility outcomes (22). In our study, AIP was not directly associated with infertility but rather might be mediated by WC. The connection between AIP and infertility, as well as the heightened risk of infertility due to WC, can be accounted for by several potential biological mechanisms. First, AIP is a biomarker of dyslipidemia. Abnormal blood lipid levels can induce IR through mechanisms involving inflammation and oxidative stress (42, 43). Dyslipidemia and IR is significantly related to WC (44, 45). IR can negatively affect oocyte quality by impairing mitochondrial function, which is crucial for maintaining oocyte health (8). Many participants with PCOS experience oxidative stress, where excess reactive oxygen species (ROS) disrupt mitochondrial function and activate inflammatory factors such as TNF-α, interleukin 1β (IL-1β), and interleukin 6 (IL-6). These inflammatory responses can impair endometrial receptivity by reducing insulin sensitivity, thereby affecting female reproductive function (46–49). Therefore, the potential mechanisms underlying the associations of AIP with WC and WC with infertility likely involve IR, dyslipidemia, oxidative stress, and inflammatory factors.

As far as we are aware, this study presents the first evidence of a stable positive link between AIP and an elevated likelihood of infertility utilizing information collected by NHANES. In light of the prospective significance of this study for future research on the link and mechanism between AIP and infertility, we think it is a valuable beginning for further study of this association. The outcomes highlighted the clinical utility of AIP as an indicator, including its capacity to replace the present, costly standard tests for diagnosing infertility and to predict the prognosis of infertility as well as the impacts of the use of assisted reproductive technologies. There should be more research done on the underlying mechanism of the link between AIP and infertility. Since these parameters are affordable, simple to use, and highly linked with the prevalence of infertility. Moreover, through mediation analysis, it is the first to confirm that WC may indeed act as a mediator in this relationship. Hence, female who attempt to conceive may enhance their likelihood of achieving pregnancy by reducing their waist circumference.

However, this study also has some limitations. First, this study was a cross-sectional study, limiting the ability to determine a causal relationship between AIP and infertility. Second, infertility outcomes were based on self-reported data, which may introduce information and recall bias and represent only the subject’s past disease state; current disease state outcomes are unknown, and these may have influenced the results. Finally, confounding factors that may have influenced the observed associations cannot be excluded.

## 5 Conclusions

The study presents the first evidence of a stable positive association between AIP and an increased risk of infertility. Additionally, it is the first to demonstrate the mediating role of waist circumference in the link between AIP and infertility. It implies that maintaining AIP within a lower range and prioritizing WC regulation is imperative to mitigate the likelihood of infertility.

## Data Availability

The datasets presented in this study can be found in online repositories. The names of the repository/repositories and accession number(s) can be found below: https://www.cdc.gov/nchs/nhanes/index.htm.
